# Tripolyphosphate Cross-Linked Macromolecular Composites for the Growth of Shape- and Size-Controlled Apatites

**DOI:** 10.3390/molecules18010027

**Published:** 2012-12-20

**Authors:** Shu-Huei Yu, Shao-Jung Wu, Jui-Yu Wu, Chih-Kang Peng, Fwu-Long Mi

**Affiliations:** 1 Department of Polymer Materials, Vanung University, Chung-Li 320, Taiwan; 2 Department of Chemical Engineering, MingChi University of Technology, Taipei 243, Taiwan; 3 Department of Biochemistry, School of medicine, Taipei Medical University, Taipei 110, Taiwan; 4 Material and Chemical Research Laboratories (MCL), Industrial Technology Research Institute (ITRI), HsinChu, 310, Taiwan; 5 Graduate Institute of Medical Sciences, College of Medicine, Taipei Medical University, Taipei 110, Taiwan

**Keywords:** chitosan, gelatin, composites, apatite, mineralization

## Abstract

Bioactive composites that enable the formation of calcium phosphates have received increased attention over the last decade, in the development of osteoconductive biomaterials for orthopaedic applications. In this work, tripolyphosphate (TPP)-cross-linked chitosan/gelatin composites (TPP-CG) were prepared for the growth of shape- and size-controlled calcium phosphates on/in the composites. The mineralization pattern of the composites, after soaking in the Ca(OH)_2_ aqueous solution, clearly demonstrated oriented, needle-like nanocrystallites of calcium phosphates in the matrix with especially high Ca/P molar ratio (3.98) as detected by energy dispersive X-ray spectroscopy (EDX) analysis. Subsequent to mineralization in a simulated body fluid (SBF), the mineralized composites showed micro-scaled spherical aggregates deposited on the surface and granule-like nanocrystallites grew in the matrix. The Ca/P molar ratio (1.72) and X-ray diffraction pattern of the nanocrystallites grown in the composites were similar to those of hydroxyapatite (HAp). Osteoblastic differentiation of ROS cells cultured on the mineralized composites allowed an enhanced expression of the chosen osteogenic marker (alkaline phosphatase, ALPase). These results indicated that the composites mineralized with micro- and nano-scaled calcium phosphates with various structural features make them attractive for bone tissue engineering applications.

## 1. Introduction

Biological materials naturally exhibit a variety of functional structures organized through a molecular self-assembly process. Bioassembled structures can provide a novel approach to fabricate advanced materials with specialized applications in numerous fields, such as drug delivery, stem cell encapsulation and tissue-engineering [1,2,3,4]. On the basis of knowledge of the synthesis of inorganic materials by naturally occurring macromolecules, bioassembled templates could be prepared to exert exquisite control over the inorganic mineralization processes for the synthesis of inorganic-organic hybrid composites [5,6]. The size, shape and polymorph of inorganic nuclei grown in the template could be controlled by the designed composites [7].

Since natural bone is a composite mainly consisting of organic and inorganic components, many efforts have been made to fabricate bone-like materials based on organic/inorganic hybridization. Several technologies such as direct mixing of the organic/apatite components, coating apatite by plasma-spraying processes and depositing apatite within the organic matrix have been developed [8,9,10,11]. It has been suggested that some proteins/peptides containing specific amino acids such as proline (Pro), glutamic acid (Glu), and phenylalanine (Phe) might be involved in the control of nucleation and growth of the mineral phase [12,13]. Additionally, a polysaccharide like chitosan was recently shown to promote the growth and deposition of the apatite crystals and was studied for use as bone substitute or tissue engineering materials [14,15,16,17].

Recent developments in biomedical research have demonstrated that nano-scaled calcium phosphates play an important role in the formation of hardened tissues in Nature [18]. Nanoparticles mineralized with calcium phosphates have a great promoting effect on the proliferation of bone marrow mesenchymal stem cells (BMSCs) [19,20]. Therefore, intensive studies have been devoted mainly towards the development of nanoapatite-based materials [21,22,23,24]. Chitosan and gelatin are both amino-containing biopolymers that have been studied for the development of biomaterials [25,26,27,28,29]. Tripolyphosphate (TPP), with its multiple phosphate groups, was able to interact with the protonated amino groups on macromolecular chains of chitosan and gelatin.

The aim of this study is to develop a functional hybrid organic-inorganic material with a bone-like apatite structure in order to accelerate bone formation. Tripolyphosphate-functionalized chitosan/gelatin composites (TPP-CG) were prepared and directed the growth of shape- and size-controllable calcium phosphates. By controlling the mineralization process, we demonstrate that mineralized calcium phosphates of variable sizes and morphologies can be observed on the surface and within the matrix of the TPP-CG composites. Osteoblastic differentiation of ROS cells cultured on the mineralized TPP-CG composites was examined by the determination of alkaline phosphatase (ALPase) activity.

## 2. Results and Discussion

### 2.1. Preparation and Characterization of the TPP-CG Composites

**Figure 1:**
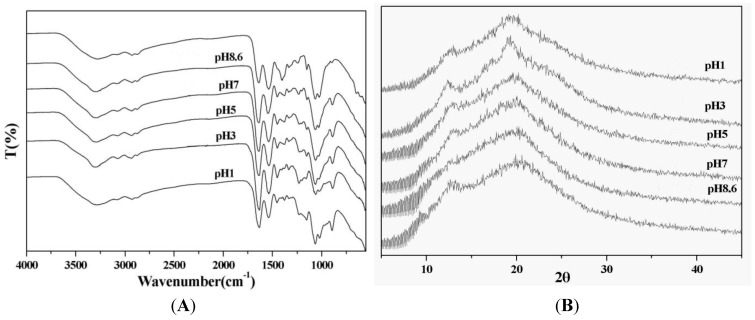
(**A**) FT-IR spectra of TPP-CG composites and (**B**) X-ray diffraction (XRD) patterns of TPP-CG composites prepared at different pH of tripolyphosphate.

Figure 1A shows the Fourier transform infrared (FT-IR) spectra of the chitosan/gelatin hybrid materials (CG composites) and the TPP-cross-linked chitosan/gelatin hybrid materials (TPP-CG composites). The CG composites were prepared at a chitosan-to-gelatin weight ratio of 1:1 due to their optimal mechanical properties (tensile strength and elongation at break, data not shown). The spectrum of the CG composites displays peaks around 905 cm^−1^ and 1,150 cm^−1^ of the assigned saccharide structure, and the characteristic amide I and amide II peaks at 1,650 cm^−1^ and 1,570 cm^−1^, respectively, corresponding to the absorption of chitosan and gelatin. The spectra of TPP-CG composites shows new peaks at around 890 and 1240 cm^−1^ which are assigned to the characteristic bands of TPP polyions (P-OH and P=O stretches). At pH 8.6, the TPP-CG composites show less obvious absorption corresponding to TPP polyions. In contrast, much stronger bands at 890 and 1236 cm^−1^ can be observed from the templates prepared using acidic TPP solutions (pH 1.0 and 3.0). This reveals that the interactions of TPP with chitosan and gelatin to form TPP-CG composites are strongly pH-dependent.

Figure 1B shows X-ray diffraction (XRD) patterns of the CG and TPP-CG composites. The peak found around 2*θ* = 20° is assigned to chitosan chains aligned through the intermolecular interactions. It is worth noting that the acidity of TPP aqueous solutions directly influences the XRD patterns of prepared TPP-CG composites. The diffraction peak around 2*θ* = 20° becomes sharper and stronger for the TPP-CG composites prepared using TPP solution of pH 3.0, as compared with its CG hybrid counterpart. It is inferred that acidic TPP mediates the assembly of chitosan and gelatin macromolecular chains and will facilitate the formation of more structured TPP-CG composites.

**Figure 2:**
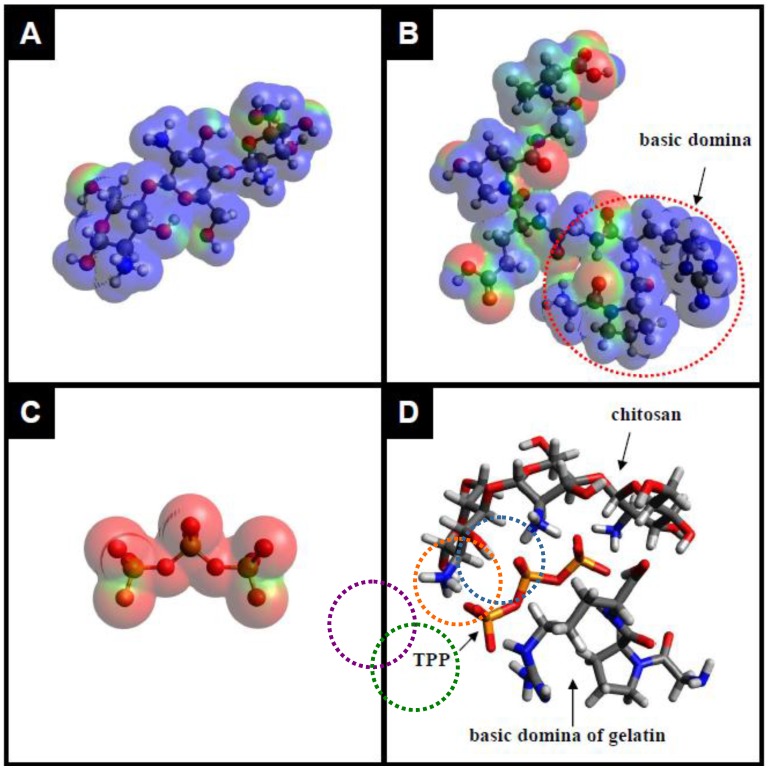
The electrostatic potential maps of chitosan (**A**), gelatin (**B**), TPP (**C**) and their electroststic interactions (**D**), showing positively charged regions in blue and negatively charged regions in red, were mapping by the Avogadro program.

Figure 2 demonstrates the electrostatic potential map of the repeating units of chitosan, gelatin, TPP and their electroststic interactions, showing positively charged regions in blue and negatively charged regions in red, mapped by the Avogadro program. It is known that the pKa value of the amino groups on chitosan is about 6.5. However, type A gelatin, with an isoelectric point of 7 to 9, is derived from collagen with exclusively acid pretreatment. At pH less than 7.0, type A gelatin would be positively charged due to its arginine-containing basic domain. In contrast, TPP is usually negatively charged under all pH conditions. At shown in Figure 2D, under acidic condition, the negatively charged TPP (mapped to red color) can interact with the protonated amino groups and arginine-containing basic domain (mapped to blue colors) on chitosan and gelatin via electrostatic attractions. It has been reported that, in hydrated form, chitosan chains are packed in an antiparallel fashion to make a sheet structure. Only on acidic condition, TPP polyions can align along the c-axis to increase the orientation of chitosan sheet and interact with the interpenetrated gelatin macromolecules, finally lead to form the TPP-CG composites.

**Figure 3:**
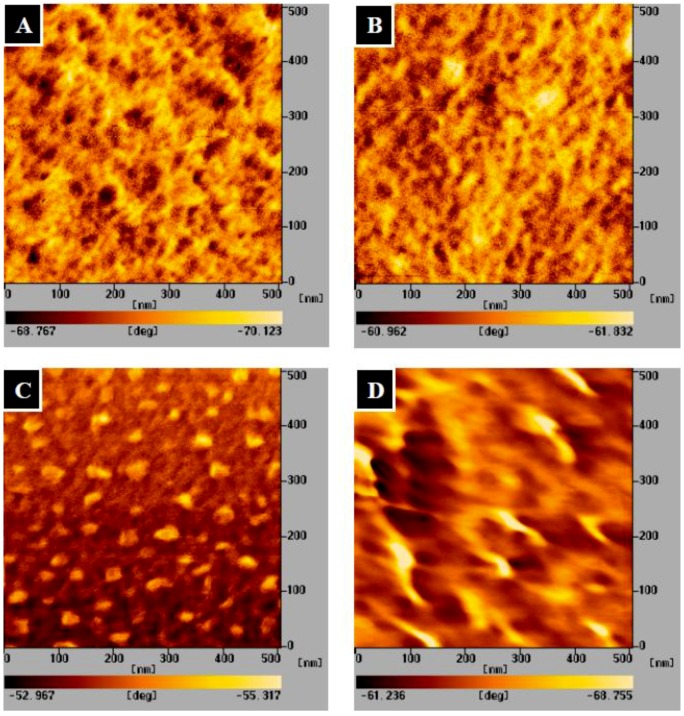
AFM images of (**A**) chitosan (**B**) gelatin (**C**) CG hybrid (**D**) TPP-CG composites.

Figure 3 shows the atomic force microscopy (AFM) images of the chitosan, gelatin, CG hybrid and TPP-CG composites. The surface phase structures of the templates can easily be identified from the height and phase images of the samples. The CG hybrid composites clearly show nano-scale (20~50 nm) phase separation (Figure 3C). The contrast in phase images is likely caused by the difference in stiffness between the separated chitosan (islands) and gelatin domain (matrix). Disappearance of the phase separation is observed from the TPP-CG composites (Figure 3D), suggesting that chitosan and gelatin biomacromolecules are assembled with TPP polyions to form inorganic-organic hybrid composites.

### 2.2. Characterization of Mineralized Calcium Phosphates

**Figure 4:**
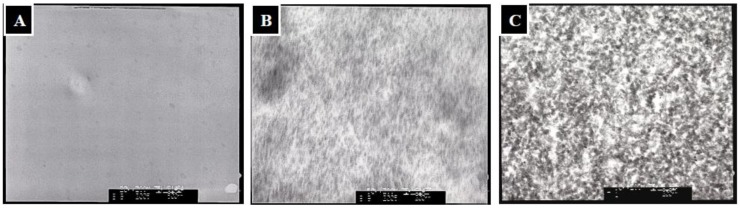
TEM micrography of (**A**) TPP-CG composites (**B**) TPP-CG composites treated with Ca(OH)_2_ (**C**) TPP-CG composites immersed in 1.5 × SBF for 21 days after pretreated with Ca(OH)_2_.

For mineralization, the TPP-CG composites were firstly immersed in Ca(OH)_2_ solution for 24 h. Afterward, the composites were washed with deionized water and immersed in 1.5 volumes of simulated body fluid (1.5 × SBF) to grow apatite for up to 21 days. The TPP-CG composites didn’t show obvious nanocrystallites in the matrix (Figure 4A). After soaking in Ca(OH)_2_, the mineralization pattern of the composites clearly demonstrated needle-like nanocrystallites in the matrix (Figure 4B). The Ca(OH)_2_ pretreated composites were then immersed in 1.5 × SBF to grow apatite for up to 21 days. The transmission electron microscopy (TEM) micrographs revealed the minerals in the composites transferred from the needle-like to granule-like nanocrystallites and were uniformly dispersed in the templates (Figure 4C).

**Figure 5:**
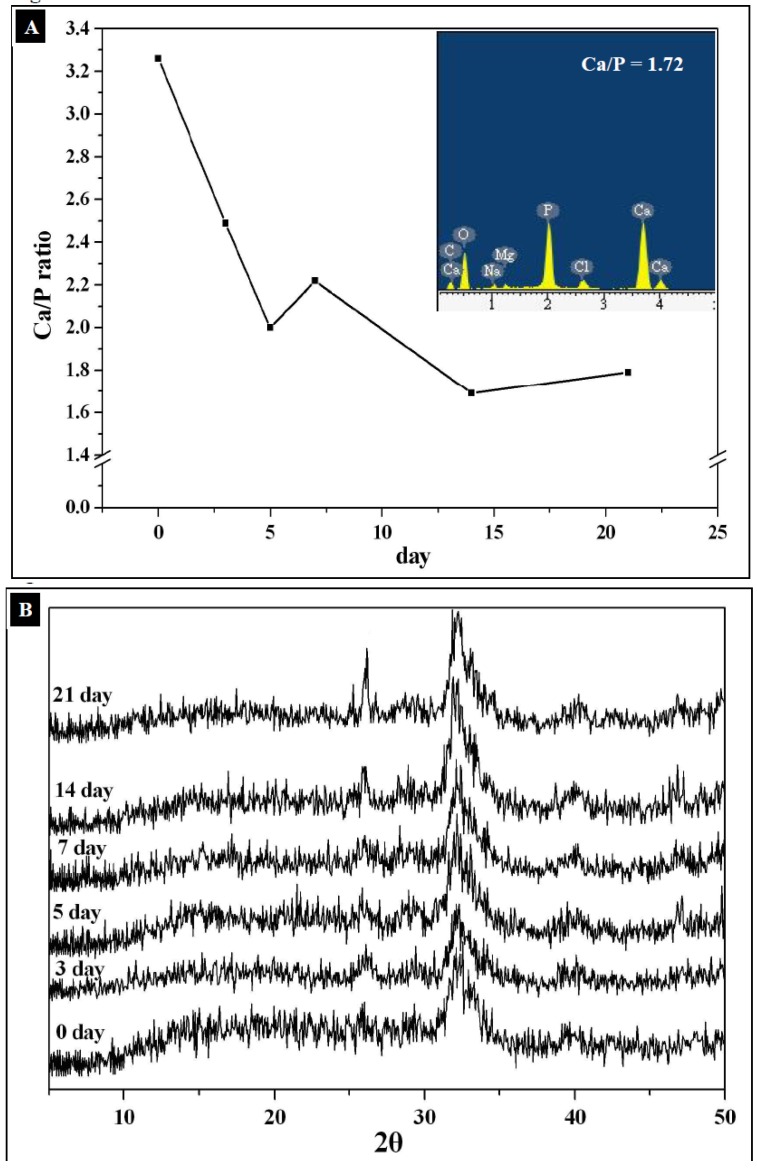
(**A**) Ca/P ratios of the mineralized TPP-CG composites determined by EDX spectra (**B**) X-ray diffraction patterns of mineral grown in TPP-CG composites.

Energy-dispersive X-ray spectroscopy (EDX) mapping of Ca and P shows that the mineral patterns are mainly composed of calcium phosphates. After immersion in 1.5 × SBF for 21 days, the Ca/P molar ratio for the nanocomposite was about 1.72 (Figure 5A), which approached the Ca/P molar ratio of HAp (Ca/P = 1.67). The X-ray diffraction patterns from these samples exhibit several reflections at 2*θ* = 26° and 32°, which correspond to the characteristic peaks of HAp (Figure 5B). EDX analysis and X-ray diffraction clearly show that the mineral phase deposited in the matrix of the template should be constituted of HAp-like materials.

The mechanism for the formation of apatite deposits proceeds through hydrolysis of the TPP polyions inside the TPP-CG composites. TPP was known to be partially hydrolyzed in aqueous solution to form phosphate ions. Treating the composites with Ca(OH)_2_ aqueous solution led to formation of a needle-like nanocrystal having especially high Ca/P molar ratio (3.28) (Figure 5A). The formation of needle-like apatite precursors with high Ca/P molar ratio was attributed to the catalytic effect of the functional groups of chitosan and gelatin which could attract a large number of calcium ions and would favor subsequent HAp nucleation.

**Figure 6:**
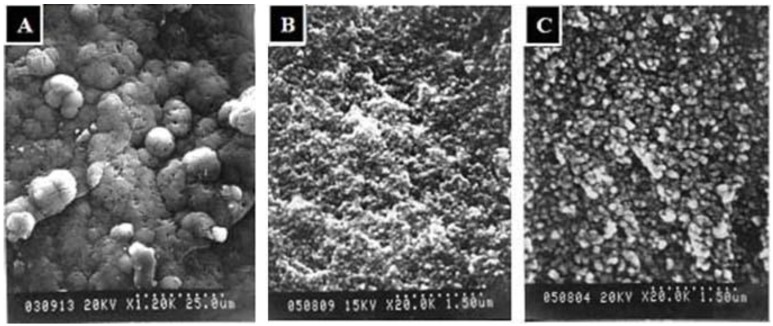
SEM investigation of mineralized templates. (**A**) mineral deposition on TPP-CG composites immersed in 1.5 × SBF for 21 days after pretreated with Ca(OH)_2_. (**B**) mineral grown in TPP-CG composites immersed in 1.5 × SBF for 3 days after pretreated with Ca(OH)_2_. (**C**) mineral grown in TPP-CG composites immersed in 1.5 × SBF for 21 days after pretreated with Ca(OH)_2_.

By soaking the Ca(OH)_2_ pre-treated composite in 1.5 × SBF, Ca^2+^ ions continuously escaped from the chitosan or gelatin macromolecular chains and were associated with PO_4_^3−^ ions to form calcium phosphates *in-situ.* Both SBF medium and the hydrolyzed TPP polyions provide the TPP-CG composites with phosphate ions for apatite mineralization. The needle-like apatite precursors were converted into granule-like HAp crystals in the TPP-CG composites after immersion in 1.5 × SBF for 21 days. SEM investigation of mineral deposition on the surface of the composite shows many micro-scale spherical aggregates (Figure 6A). The Ca/P molar ratio detected by EDX on the surface of the composite is about 1.89, suggesting that the composition of the spherical aggregates is based on calcium phosphate with relatively higher Ca content than HAp.

The formation of spherical aggregates of calcium phosphates can be attributed to the strong affinity between calcium ions and the functional groups on the surface, leading to the 2-dimensional outward growth of micro-scale calcium phosphate. The spherical aggregates of calcium phosphates demonstrated relatively high Ca/P ratios because they lack hydrolyzed TPP polyions on the surface of the templates. Scanning electron microscope (SEM) investigation of the mineralized TPP-CG composites (cross-section) showed different sizes of mineral nanoparticles deposited in the matrix of the composite after 3 days (~50 nm) and 21 days (~150 nm) of mineralization (Figure 6B,C). However, the sizes of those nanoparticles grown in the composite are smaller than the spherical aggregates (5~20 μm) deposited on the surface (Figure 6A). The size and shape change by TEM (granule-like nanocrystallites, Figure 4C) and SEM (micro-scaled spherical aggregates, Figure 6A) is due to the different mechanism in crystallization of calcium phosphate respective on the surface and in the matrix of the TPP-CG composite templates, due to limited crystal growth by the polymer matrix. The mineralized nanoparticles have been previously characterized by EDX mapping and X-ray diffraction to shows that it should be constituted of HAp-like materials (Figure 5A,B).

**Figure 7:**
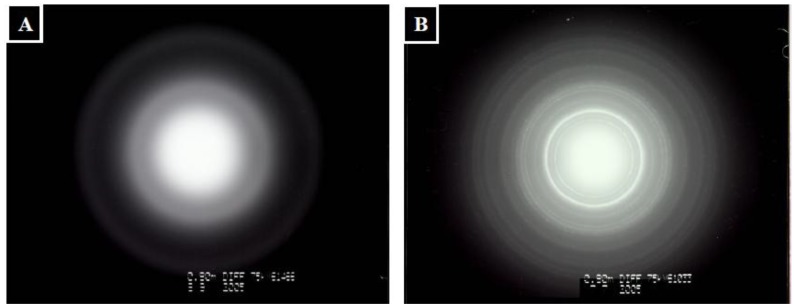
TEM electron diffraction patterns of (**A**) original TPP-CG composites and (**B**) mineral deposition on TPP-CG composites immersed in 1.5 × SBF for 21 days after pretreated with Ca(OH)_2_.

Figure 7A and Figure 7B shows the TEM electron diffraction patterns of the original and mineralized TPP-CG composites. HAp within the mineralized composites exhibits inclines to grow in the c-axis [29], which corresponds to the (0 0 2) reflection. Based on the above considerations, the (0 0 2) reflection can be used to monitor the HAp formation. As shown in Table 1, after pretreatment with Ca(OH)_2_, the sample showed no (0 0 2) reflection peak, suggesting that HAp was still not grown in the composites. After soaking in 1.5 × SBF, the crystallinity and average crystallite sizes of HAp, as calculated by Scherer’s equation, are growing as the reaction time increases. The crystallinity values were found to be less than one, indicating a low crystallinity of the nanoapatite. It is suggested that in natural bone synthesis, the biomineralization process starts with the formation of poorly crystalline calcium apatites [30]. Moreover, pure ceramics with high crystallinity are reported to be nonbioresorbable materials and could only serve as permanent implants *in vivo* [31]. The average crystallite sizes calculated by Scherer’s equation were found to be smaller than the real size of HAp, as observed from TEM micrographs. The deviation of the average crystallite sizes calculated by Scherer’s equation is due to the interference from chitosan and gelatin macromolecular chains of the composites.

### 2.3. Measurement of ALPase Activity

**Figure 8:**
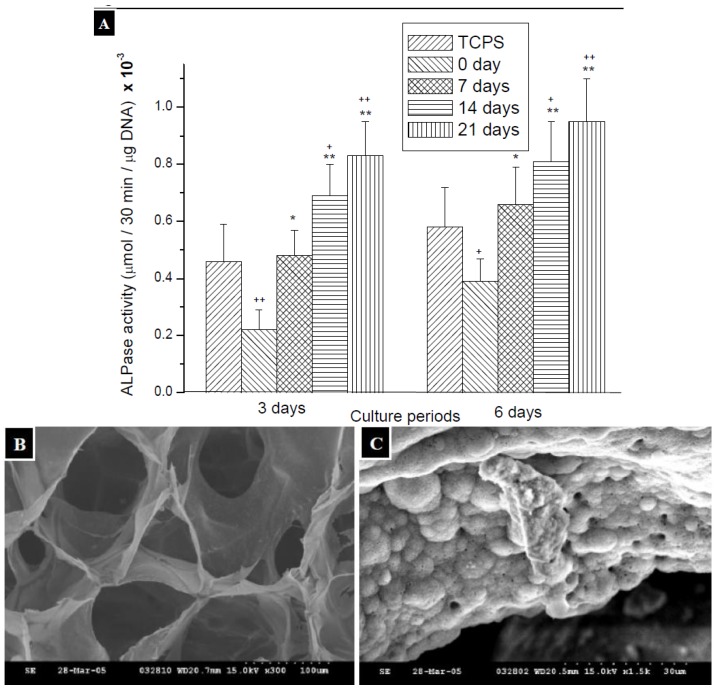
(**A**) The changes of ALPase activity in ROS cells cultured with TCPS and chitosan films which were treated with biomimetic body fluid for 0 days, 7 days, 14 days and 21 days. Significance (*t*-test) is indicated: * *p* < 0.05, ** *p* < 0.01 compared with 0 days films for the same culture time, ^+^ *p* < 0.1, ^++^ *p* < 0.05 compared with TCPS for the same culture time (n = 4). (**B**) SEM micrography of the porous TPP-CG scaffold. (**C**) SEM micrography on the wall of the pores of the TPP-CG scaffold mineralized with calcium phosphates.

ALPase is a representative enzyme of osteoblastic differentiation, and then ALPase activity was determined as an indicator of osteoblastic differentiation of ROS cells. As the ALP activity increased during the process of culture with materials, the calcification of osteoblastic cells would be stimulated [32]. As shown in Figure 8A, the ALPase activity of ROS cells by 6 days of culture is higher than that by 3 days of culture, which corresponded to the results in previous researches showing that the ALPase activity of ROS cells would increase with culture time in the initial culture period [33]. For the ROS cells cultured with mineralized TPP-CG composites, ALPase activity was increased gradually by the time of treatment with 1.5 × SBF, no matter whether there were cultured for 3 days or 6 days. Besides, it was interesting to note that ROS cells grown on TPP-CG composites, which have been mineralized for 7 days, exhibited an ALPase activity that was comparable to the cells on TCPS. The ALPase activity is higher than the control groups, the ROS cells cultured on TCPS, with the treatment for 14 and 21 days. The results suggested that the TPP-CG composites are more effective on osteoinduction by the treatment of 1.5 × SBF, so the of expression of ALPase of ROS cells, osteoblastic cells, is significant enhanced. The enhancement is even more notable with longer time of treatment with 1.5 × SBF and the effect would be comparable to the commercial TCPS. In this study, it has been found that the deposition of various apatite-like materials on/in TPP-CG composites can regulate the expression of ALPase, the marker attributed to the osteoblastic phenotype. The TPP-CG composites can also be reshaped to macroporous scaffolds by freeze-drying (Figure 8B,C) therefore can be used as biomimetic mineralized, multi-component scaffolds for bone tissue regeneration.

## 3. Experimental

### 3.1. Materials

Gelatin (acid extracted from bovine skin with bloom number equal to 225) was a product of Sigma Chemical Co. (St. Louis, MO, USA). Chitosan with a degree of deacetylation of approximately 85% were acquired from Fluka Chemical Co. (Buchs, Switzerland). Sodium tripolyphosphate (TPP) was purchased from Showa Chemical Co. (Tokyo, Japan). All other reagents and solvents used were of reagent grade. The reagents for the measurement of ALPase, including *p*-nitrophenylphosphate, MgCl_2_, ALPase and DNA quantification kit, were purchased from Sigma Chemical Co.

### 3.2. Preparation of TPP-CG Composites

Tripolyphosphate-functionalized chitosan/gelatin composites (TPP-CG) were prepared using a casting/in-liquid curing technique. Firstly, chitosan was blended with gelatin as follows: a stock solution of chitosan in water (1.5%, w/v) was prepared by dissolving chitosan (0.3 g) in aqueous acetic acid (20 mL, 0.5%, w/v) and stirring for 12 h at room temperature. The chitosan-to-gelatin weight ratios in the mixtures were 1:1. Subsequently, appropriate amounts of gelatin, as per the aforementioned chitosan-to-gelatin weight ratios, were added to this stock solution. The chitosan-to-gelatin weight ratios in the mixtures were 1:1. After thoroughly stirring, the air-bubble-free solutions were poured into shallow dishes and dried in air at room temperature for 12 h to prepare chitosan-gelatin hybrid films (CG composites). The hybrid CG composite films were pre-swelled with deionized water then soaked in TPP aqueous solutions (10% w/v) at 4 °C for 2 h, to prepare the TPP-CG composite films. Because 0.2 g of the hybrid CG composite film containing 4.88 × 10^−3^ mol amino groups/g (gelatin contains 0.33 × 10^−3^ mol amino groups/g and chitosan contains 4.55 × 10^−3^ mol amino groups/g) were reacted with 25 mL, 10% w/v TPP (3.39 × 10^−2^ mol phosphate groups/g), the molar ratio between free amine groups of the two polymers and the TPP was 1:6.95. The pH values of TPP solutions were kept at 1.0, 3.0, 5.0, 7.0 and 8.6, respectively. Finally, the TPP-CG composites were rinsed with deionized water for 12 h at 4 °C to remove residual TPP and were dried in air for 12 h.

### 3.3. Characterization of the TPP-CG Composites

FT-IR analysis was conducted by mixing the ground powder of TPP-CG composites with KBr (1:100). The mixed powder then was pressed into a disk and analyzed by a FT-IR spectrometer (Perkin Elmer Spectrum RXI FT-IR System, Buckinghamshire, U.K.). The X-ray diffraction patterns of the TPP-CG composites were recorded using a Shimadzu XD-5 diffractometer with Cu Kα radiation. Atomic force microscopy (AFM, Dimension 3100, Nanoscope IV Digital Instruments, Santa Barbara, CA, USA) was used to observe the morphology and roughness of the TPP-CG composites.

### 3.4. Biomineralization

The TPP-CG composites were soaked in freshly prepared saturated Ca(OH)_2_ solution for a period of 24 h. After treatment, the templates were thoroughly washed with distilled water and then soaked in simulated body fluid (SBF). The SBF with modified formulation (1.5 × SBF) were prepared as described previously by dissolving reagent grade NaCl, NaHCO_3_, KCl, K_2_HPO_4_·3H_2_O, MgCl_2_·6H_2_O, CaCl_2_, and Na_2_SO_4_ in deionized water. The films were retrieved after 3, 7, 14 and 21 days of soaking. The retrieved samples were thoroughly rinsed with distilled water and then dried in air.

### 3.5. Characterization of Mineralized Calcium Phosphates on/in the Biotemplate

The morphology of the nanocrystals growth within the TPP-CG composites were directly examined by transmission electron microscopy (TEM, Hitachi H-7100, Tokoy, Japan). Additionally, the TPP-CG composites were also examined by a scanning electron microscope (SEM, Hitachi S-2400). The mineralized TPP-CG composites were cut with a razor, attached onto a double-sided adhesive tape and fixed to an aluminum stage. Subsequently, the templates were sputter-coated with gold in a thickness of 500 × 10^−8^ cm using a Hitachi coating unit (IB-2 coater). The morphologies of the mineralized templates (surface and cross-section) and the energy profiles of Ca/P ratios were examined using the Hitachi S-2400 SEM equipped with an attachment of EDX analyzer (Kevex Delta 80000). The x-ray diffraction patterns of mineralized calcium phosphates on/in the biotemplate were recorded using a Shimadzu XD-5 diffractometer with Cu Kα radiation.

### 3.6. Determination of Crystallites Size of Hydroxyapatite (HAp)

The crystallite size of HAp within the TPP-CG composites were calculated by Scherer’s equation [17]:

L = *K*λ/*β_m_* cosθ

where ***L*** is the average crystallite size, *β_m_* the full width of the peak at half of the maximum intensity (rad), λ the wavelength of X-ray radiation (1.54178 Å), *K* is a constant related to the crystallite shape and is approximately equal to unity.

The crystallinity (*X_c_*) was deduced, according to the following equation:

$$\beta_{002}\times \sqrt[{3}]{X_{c}}=K_{A}$$

where *X_c_* is the crystallinity degree, *β*_002_ the full width of the peak at half intensity of (0 0 2) reflection in (degree-2θ), K_A_ is a constant set at 0.24.

### 3.7. Measurement of ALPase Activity

The ROS 17/2.8 cells cultured for 3 days and 6 days on the TCPS films and mineralized TPP-CG composites are dissolved, respectively, with 0.05N NaOH. The solution of cell layer was used as an enzyme solution for determination of ALPase activity based on the method of Hou *et al*. [33]. The reaction was continued for 30 minutes, and measured at 405 nm with spectrophotometer (Beckman-DU640, Brea, CA, USA).

Briefly, at indicated test periods, cells were initially washed with PBS and then scraped out of the dishes with a rubber policeman into the ice-cold Tris-HCl buffer (10 mM, pH 7.4). The harvested cells were sonicated in 1 mL of 10 mM Tris-HCl buffer for 15 sec in an ice bath. Then 0.2 mL sonicates were added to ALPase assay buffer (0.6 mL) consisting of 0.05bM sodium carbonate (pH = 10.2), 2 mM MgCl_2_, 2 mM *p*-nitrophenylphosphate (pH = 10.5). The reaction mixture was incubated for 30 min at 37 °C and the reaction was stopped by adding 0.2 mL of 2N NaOH. ALPase activity was measured by absorbance at 410 nm and read by Beckman DU-65 spectrophotometer. Enzyme activities of ALPases were expressed as μmol *p*-nitrophenolphosphate/30 min/μg DNA.

## 4. Conclusions

In summary, we have demonstrated the TPP-mediated supramolecular assembly of chitosan-gelatin composites (TPP-CG composites)**.** The templates possess the ability to mineralize various calcium phosphates. The size, shape and Ca/P molar ratio of the calcium phosphates deposited on the surface and grown in the matrix of the templates could be controlled. ROS cells grown on the mineralized TPP-CG composites exhibited a general trend of increased ALPase activity with increasing time for the treatment with 1.5 × SBF.

## Figures and Tables

**Table 1:** The average crystallite sizes calculated by Scherrer’s equation.

Time (day)	Line width (002) FWHM (°)	Cristallinity (Xc)	Line width (002) FWHM (rad)	Average crystallite size, L (nm) by Scherrer’s equation
0	-	-	-	-
3	0.98536	0.0144	0.0172	9.2
5	0.86683	0.0212	0.0151	10.5
7	0.76252	0.0312	0.0133	11.9
14	0.52156	0.0974	0.0091	17.4
21	0.44592	0.1559	0.0078	20.3
